# Effectiveness of acupuncture intervention for neck pain caused by cervical spondylosis: study protocol for a randomized controlled trial

**DOI:** 10.1186/1745-6215-14-186

**Published:** 2013-06-22

**Authors:** Qinghui Que, Xiaode Ye, Quangui Su, Yan Weng, Jianfeng Chu, Lijuan Mei, Wenwen Huang, Renhui Lu, Guohua Zheng

**Affiliations:** 1 Affiliated Rehabilitation Hospital, Fujian University of Chinese Medicine, Fuzhou 350002, China; 2 Academy of Integrative Medicine, Fujian University of Traditional Chinese Medicine, Fuzhou 350122, China; 3 Rehabilitation Medicine College, Fujian University of Traditional Chinese Medicine, Fuzhou 350002, China

**Keywords:** Acupuncture, Efficacy and safety, Neck pain caused by cervical spondylosis, Randomized double-blind placebo-controlled trial

## Abstract

**Background:**

Neck pain caused by cervical spondylosis has become a common health problem worldwide among >40-year-old adults. Acupuncture intervention is one of the most popular treatment measures for this disorder. However, evidence for its efficacy in relieving neck pain and recovering neck physiological function has not been established in randomized, placebo-controlled trials. The primary aim of this trial is to assess the efficacy and safety of active acupuncture compared with sham acupuncture intervention for neck pain caused by cervical spondylosis.

**Methods/Design:**

We will conduct a randomized, double-blind, parallel-group, placebo-controlled trial comparing active acupuncture with placebo (sham acupuncture). A total of 456 patients with neck pain caused by cervical spondylosis who meet the eligibility criteria from outpatient clinics of the Second People’s Hospital of Fujian Province and the Affiliated Rehabilitation Hospital, Fujian University of Traditional Chinese Medicine will be recruited and randomized into an active acupuncture or sham acupuncture group. The participants will undergo treatment sessions with either active or sham acupuncture intervention five times a week for 2 weeks. Evaluation by blinded assessors at baseline and at intervention for 1 and 2 weeks will include demographic characteristics, validated questionnaires (Northwick Park Neck Pain Questionnaire (NPQ) scale, Short-Form 36 (SF-36) scale, and McGill pain scale), examination of neck physiological function, and adverse events. All included patients will be followed up and investigated for relapse of neck pain at 4, 8, and 12 weeks after intervention.

**Discussion:**

This paper describes the rationale and design of a randomized double-blind, placebo-controlled trial that aims to determine the efficacy and safety of acupuncture intervention for neck pain caused by cervical spondylosis. The primary outcomes are changes in the NPQ score and neck physiological function. Secondary outcome measures include quality of life, adverse events, and relapse of neck pain. If successful, this project will provide evidence of the efficacy and safety of acupuncture for neck pain caused by cervical spondylosis.

**Trial registration:**

Chinese Clinical Trial Registry: ChiCTR-TRC-12002206.

Registration date: 11 May 2012.

## Background

Cervical spondylosis (CS) is defined as age-related chronic disc degeneration. It is also defined as vertebral osteophytosis secondary to degenerative disc disease, which in the cervical spine may be asymptomatic or can present as pure axial neck pain, cervical radiculopathy, cervical myelopathy, or cervical myeloradiculopathy [1,2]. Neck pain caused by CS (CS neck pain) is associated with slight degenerative changes within the intervertebral disc in early CS [3]. CS is usually asymptomatic, but may present with symptoms of neck pain, neck stiffness, or even shoulder pain and stiffness. Its etiological factors are multifactorial and involve poor posture, anxiety, depression, neck strain, and sporting or occupational activities [4]. With acceleration of the pace of modern life, computers, air conditioning, fans, and cars have become widely used, and CS neck pain has become a common health problem worldwide. It is reportedly associated with a 50% incidence of radiological evidence in individuals over the age of 40 years and an 85% incidence of radiological evidence in individuals over the age of 60 years [5]. Neck pain is one of the most frequent issues among patients with CS. About two-thirds of adults in the UK reportedly experience neck pain at some time in their lives, with men more commonly affected than women, at a ratio of 3:2 [6]. A Norwegian survey of 10,000 adults showed that 34% of respondents had experienced neck pain in the previous year [7]. In Hong Kong, the prevalence of neck pain caused by CS is 15% to 17%, and the lifetime prevalence is 30% to 50% [8]. Approximately 33% of adults in Canada and 43% in Sweden had experienced neck pain in the past several months [9,10].

Non-surgical treatment is usually the most appropriate course of initial management. Most CS neck pain responds to conservative measures. Stress management or postural advice in daily activities, work, and hobbies is effective for some patients with slight neck pain. The current clinical treatments mainly include administration of non-steroidal anti-inflammatory drugs, muscle relaxants, physiotherapy, analgesics, and so on [11]. However, there is little evidence to support the efficacy of these therapies for CS neck pain. The most optimal treatment has not yet been established.

Traditional Chinese medicine measures, such as acupuncture, massage, neck exercises and so on have been extensively used for the management of CS neck pain in China and other countries; among these, acupuncture is one of the most popular measures. A number of clinical studies have been conducted to evaluate its efficacy [12-16]. Their results suggested that acupuncture is effective for pain relief in the immediate and short-term post-treatment period; however, the efficacy of a mid-term to long-term benefit for pain control or functional improvement was less clear [17-19]. Furthermore, comparative trials in the literature are few in number and of poor quality. Therefore, we will conduct a rigorous evaluation to test whether acupuncture performs better than placebo for treatment of CS neck pain.

The overall goal of this project is to determine the relative effectiveness and safety of acupuncture for CS neck pain.

## Methods/design

### Study design

This study will be a randomized, double-blind, parallel controlled clinical trial to evaluate the effectiveness and safety of acupuncture for CS neck pain. A total of 458 subjects with CS neck pain from outpatient clinics will be recruited and randomly allocated to either the active acupuncture group (experimental group) or the sham acupuncture group (control group). The Second People’s Hospital of Fujuan and the Affiliated Rehabilitation Hospital, Fujian University of Chinese Medicine will be responsible for recruiting, screening, and intervention of all patients and assessment of all outcomes. Management of the randomization sequence, blinding, and data analyses will be carried out by the Center for Evidence Based Medicine of Fujian Traditional Chinese Medicine University. Ethics approval has been received from Fujian Traditional Chinese Medicine University. Informed written consent will be obtained from all participants.

### Participants

**Figure 1:**
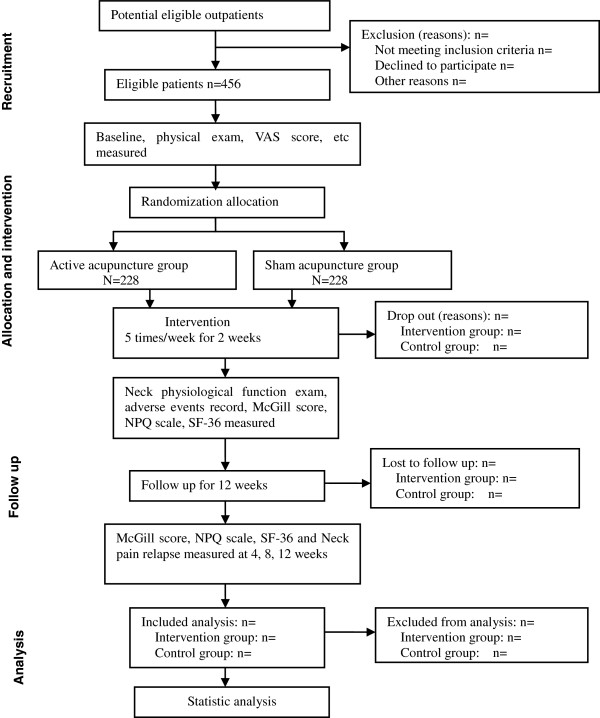
Flow diagram of participants.

We will include patients between 18 and 60 years of age with CS neck pain. Patients must have typical symptoms of neck pain and stiffness, but their cervical vertebrae must be normal or show only physiological curvature changes on radiographic examination. Potentially eligible participants are being identified through screening of patient medical records at the participating hospitals. Screening of patients will be performed by one of the authors according to the inclusion and exclusion criteria. A flow diagram of the patients is shown in Figure 1.

### Inclusion criteria

To be eligible, participants must fulfill the following criteria: (1) a confirmed diagnosis of CS neck pain in accordance with the diagnostic criteria published by the Chinese Association of Rehabilitation Medicine (2010) [20] and with reference to the *International Classification of Diseases,* 10th edition (ICD-10) codes (http://www.icd10data.com): M47.812 (other spondylosis without myelopathy or radiculopathy (cervical region)); (2) male or female 18 to 60 years of age; (3) with/without positive signs of musculoskeletal system upon physical examination; (4) pain intensity ranging from 3 to 7 points on a visual analog scale (VAS) upon recruitment; (5) no acupuncture or other relative treatment within the last 7 days; and (6) informed consent document has been signed.

### Exclusion criteria

The exclusion criteria were as follows: (1) history of neck trauma or T1 to T6 thoracic vertebra trauma; (2) has vertebral body or spinal canal cancer, tuberculosis, or severe osteoporosis; (3) history of neck surgery or presence of congenital malformation of the cervical vertebrae; (4) pregnant or lactating (women) or presence of a mental disorder; (5) has a severe systemic disease such as cardiocerebrovascular disease, tumors, diabetes mellitus, kidney disease, or digestive system disease; (6) inability to complete research questionnaires; and (7) rejection of randomization.

### Sample size

The sample size calculation was based on a reference of a similar intervention in similar patient groups using the same primary outcome instrument [21]. The primary outcome measure is improvement of scores in neck pain as measured with the Northwick Park Neck Pain Questionnaire (NPQ) at the end of treatments. According to previous research [21], the mean and standard deviation of NPQ scores were (24.04 ± 11.83) in the placebo group and (20.71 ± 11.91) in the acupuncture group. The sample size was calculated based on the formula:

(1) $$n_{1}=n_{2}=2{\left(\frac{z_{\alpha /2}+z_{\beta }}{M_{e}-M_{c}}\right)}^{2}\delta^{2}$$

with a type I error of 5% (α = 0.05) and 80% power (β = 0.2). A total of 198 patients are required in each group to show statistical differences between the groups. We estimated that 15% patients will drop out of the study; thus, 456 patients was established as the sample size.

### Randomization and allocation

The randomization schedule will be prepared by the Evidence-Based Medicine Center of the Academy of Integrative Medicine, Fujian University of Traditional Chinese Medicine. The specific randomization lists will be computer-generated using the SAS 8.12 software PLAY PLAN program (SAS software (Beijing) Co., Ltd, No. 1 Changan Road, Dongcheng District, Beijing , China) and concealed from the screeners, assessors, and patients by a designer from the Evidence-Based Medicine Center who is not involved in the study. Cards with group assignments and detailed treatment schedules will be prepared by the Evidence-Based Medicine Center and sealed in opaque envelopes. These sealed envelopes will be marked with the patients’ sequential numbers and kept by the screeners who are responsible for the screening of participants. When a patient has been enrolled, written informed consent has been obtained, and the patient has completed the baseline measurements and is confirmed to be an eligible participant, the envelope corresponding to the sequential number of the patient will be transmitted to the acupuncturist. The acupuncturist will open the envelope and perform the intervention (active or sham acupuncture) according to the treatment schedule written on the card. The information of the allocation list will remain confidential and kept by the acupuncturist not involved in participant recruitment or outcome assessment, and this information will not be shared with the data analyzers or outcome assessors.

### Blinding

The outcome assessors will be blinded to the randomization allocation and will not be involved in performance of the interventions. Participants will be blinded to the group allocation and will not know whether they have been treated with active or sham acupuncture. It will be impossible to blind the acupuncturists because they will perform the interventional protocols. However, the acupuncturists will not take part in the outcome measurements or the statistical analyses, and will be requested not to disclose details about their treatment to the outcome assessors or participants. The statistician will be blinded to the group allocation until completion of the statistical analyses.

### Interventions

The intervention scheme originates from the guidelines of diagnosis, treatment, and rehabilitation for CS [20] and has been practiced for some time in the Second People’s Hospital of Fujian Province and the Affiliated Rehabilitation Hospital, Fujian University of Chinese Medicine. We hypothesized that acupuncture for CS neck pain would have a more positive effect in terms of relieving neck pain and recovering neck physiological function than placebo (sham acupuncture). Therefore, participants in the experimental group will receive the active acupuncture treatment, and participants in the control group will receive the sham acupuncture treatment. All participants will receive 25 mg of diclofenac sodium three times a day if their VAS scores exceed 5.

### Active acupuncture

Active acupuncture treatment will be applied to the participants in the experimental group. Active acupuncture stimulation will be performed at the acupuncture points *Jing-jiaji* (EX-B2.C5), *Jing-jiaji* (EX-B2.C6), *Jing-jiaji* (EX-B2.C7), and *Jianzhongshu* (Sl15) on both sides of the body [22]. Sterile single-use acupuncture needles (40 mm in length and 0.30 mm in diameter; Hwato brand, Suzhou Medical Products Factory Co., Ltd., 12–14 West Qilin lane, Suzhou, China) will be perpendicularly inserted to a depth of 20 to 30 mm at the selected stimulation point. When the treated participants achieve *de qi* sensation (soreness, numbness, distention, and heaviness), electroacupuncture with its anode connected to *Jing-jiaji* (EX-B2.C5) and cathode connected to *Jing-jiaji* (EX-B2.C7) will be performed to stimulate the relevant acupuncture points for 20 minutes by a single experienced acupuncturist using a continuous-wave, 1-Hz frequency and a comfortable strength.

### Sham acupuncture

Sham acupuncture treatment will be applied to the participants in the control group. The operation of sham acupuncture will be same as that of active acupuncture with the exception of the sites and depth used. Sham acupuncture stimulation will be performed at sites 1.5 cm away from *Jing-jiaji* (EX-B2.C5), *Jing-jiaji* (EX-B2.C6), *Jing-jiaji* (EX-B2.C7), and *Jianzhongshu* (Sl15), and the depth of the inserted needles will be controlled within 15 mm. The operation of electroacupuncture in the control group will be same as that in the experimental group.

### Intervention regimen

Patients will be required undergo ten treatment sessions at a frequency of five sessions a week, and the intervention will be completed in 2 weeks. All electroacupuncture procedures will be performed with a Hwato brand SDZ-II electronic needle therapy instrument made by Suzhou Medical Products Factory Co., Ltd. The two groups will be separated from the treatment, which will be performed by a single acupuncturist.

### Follow-up period

During the 12-week unsupervised follow-up period, no participants will undergo special therapy with the exception of routine cervical care. At post-treatment weeks 4, 8, and 12, the outcome assessor will telephone participants to investigate the recurrence of neck pain.

### Outcome measurement

The following baseline descriptive data will be obtained by questionnaire and physical examination: age, sex, marital status, education level, employment status, family history of CS, VAS score of cervical pain, function of cervical vertebrae, and previous health problems. The radiographic severity of cervical vertebral changes will be assessed on X-ray images. A summary of all measures in the trial is shown in Table 1.

**Table 1:** Study assessments, procedures, and timetable

**Items**	**Before grouping**	**First intervention period (5 days/week)**		**Second intervention period (5 days/week)**		**Follow-up Period**		
Time point (day)	0	1 to 7	7	8 to 14	14	42	70	98
Case diagnosis	X							
Inclusion criteria	X							
Exclusion criteria	X							
Informed consent	X							
Baseline information log	X							
Physical examination	X							
Visual analog scale score	X							
Randomization allocation	X							
Neck physiological function examination		X		X				
Intervention and record		X		X				
Adverse events record		X		X				
McGill pain score			X		X			
Northwick Park Neck Pain Questionnaire score	X		X		X			
Short-Form 36 measure	X		X		X			
Neck pain relapse					X	X	X	X

### Primary outcome measures

The primary outcomes are changes in the symptoms of CS and function of the cervical vertebrae. Symptoms of CS will be measured using the NPQ [22]. Function of the cervical vertebrae, mainly including the activity of the cervical vertebrae upon forward bending, lateral bending, and rotation, will be measured using physical examination and assessed by the outcome assessors.

### Secondary outcome measures

Quality of life will be assessed using the Short Form-36 (SF-36) Questionnaire [23]. Neck pain will be assessed using the McGill Pain Questionnaire [24]. The recovery period is defined as the number of days from the beginning of treatment to recovery of cervical physiological function and will be recorded by the outcome assessors. Relapse of neck pain will be recorded by telephone investigation during the follow-up period.

### Safety evaluation

Any adverse events (defined as any functional lesion caused by the interventions, such as local bleeding at the needle insertion point; local numbness, pain, or dizziness during treatment and so on) will be recorded during treatment. If any adverse event occurs, the doctor will provide the corresponding treatment to the patient. The adverse events will be immediately reported to the primary investigator and ethics committee to decide if the patient needs to withdraw from the trial.

### Data collection and management

The demographic and baseline characteristic data will be collected by screeners when the patients are recruited. Clinical outcome measurement and questionnaire-based assessment of treatment effects and neck physiological function will be measured by the outcome assessors after the treatment is completed. During the follow-up period, relapse of neck pain will be investigated by a telephone call at 4, 8, and 12 weeks post treatment.

Research assistants will conduct quality control of data collection and be responsible for data entry. The data manager will be responsible for initial data cleaning, identifying, coding, and conversion into the proper format for data analysis.

### Statistical analysis

The primary outcomes will undergo intent-to-treat analysis based on the initial treatment assignment and focus on assessment of the main effects of the acupuncture treatment for CS at symptoms of CS and function of the cervical vertebrae. Analyses will be performed using IBM SPSS 20 (version 20.0, IBM Corp., New York, NY, USA). The continuous variables and descriptive values will be expressed using means with standard deviations or medians with ranges. For the variables with a normal distribution, statistical comparisons between the groups will be made by using a *t* test. If the variables have a non-normal distribution or ordinal level, statistical comparison between groups will be made using the Mann–Whitney *U* test. Measures with a discrete distribution will be expressed as percentages and analyzed by the *χ*^2^ or Fisher’s exact test as appropriate. The amount of extra diclofenac sodium used between the experiment and control groups will be analyzed, and a general linear model will be applied to adjust its confounding influence if necessary.

## Discussion

In this randomized, double-blind controlled trial of patients with CS neck pain, we intend to investigate the mid-term efficacy and safety of acupuncture for CS neck pain. This is a well designed study in strict accordance with the Consolidated Standards of Reporting Trials (CONSORT) statement of randomized controlled trials and will enable determination of the effects of acupuncture [25]. First, we estimated the most optimal sample size to ensure adequate test performance. Second, we ensured that this study is truly a randomized controlled trial to the greatest extent possible by full implementation of randomization and blinding. In addition, to assess the efficacy of acupuncture for CS neck pain, the placebo effect should be avoided. At present, ‘sham acupuncture’ is considered to be the most commonly proposed placebo control. In this protocol, sham acupuncture is applied by adapting the depth of needling at non-acupuncture points. This technique has been widely used in clinical trials, many of which have adopted this method as a control [26-30]. Its main advantages are that it is the strongest type of patient blinding and is easy to carry out. Its main problem is that the control group may play a role in the treatment of the disease; thus, the effect of acupuncture maybe be underestimated [31]. However, if the experimental results prove that acupuncture is indeed better than the control group treatment, the efficacy of acupuncture for CS neck pain will be positive. In terms of evaluation indicators, we will use a reasonable specification, design a rigorous professional scale as the evaluation indicator of the primary outcome, and avoid a subjectively developed fuzzy classification evaluation, distorting data less and reducing bias. The interventions (active or sham acupuncture) will be performed by a fixed chief physician, who has more than 20 years of clinical experience. This will improve the participants’ compliance and reduce the risk of dropout.

In summary, we have combined a proper clinical trial design and acupuncture features and finished a scientific design. We will report the clinical trial results in accordance with international norms for randomized controlled trial reporting (CONSORT) [25] and acupuncture clinical trial intervention reporting standards (Standards for Reporting Interventions in Clinical Trials of Acupuncture (STRICTA)) [32]. This study has also been pasted by the Ethics Committee of Fujian University of Traditional Chinese Medicine; consequently, the results of this study will generate scientifically rigorous clinical evidence for the study of CS.

## Trial status

Ongoing recruitment.

## Competing interests

The authors declare that they have no competing interests.

## Authors’ contributions

QQH, ZGH, and YXD conceived of the study, designed the study protocol, and drafted the manuscript. ZGH and QQH revised study protocols and wrote sections of the manuscript. QQH is in charge of coordination and direct implementation. SQG, WY, HWW, CJF, MLJ, and LRH helped to develop the study measures and analyses. All authors contributed to drafting the manuscript and have read and approved the final manuscript.
